# The Underrated Salivary Virome of Men Who Have Sex With Men Infected With HIV

**DOI:** 10.3389/fimmu.2021.759253

**Published:** 2021-12-02

**Authors:** Ying Guo, Xiaojie Huang, Xintong Sun, Yixi Yu, Yan Wang, Baojin Zhang, Jie Cao, Shuo Wen, Yuchen Li, Xin Wang, Siyu Cai, Wei Xia, Feili Wei, Junyi Duan, Haozhi Dong, Shan Guo, Fengqiu Zhang, Dongxiang Zheng, Zheng Sun

**Affiliations:** ^1^ Department of Stomatology, Beijing Youan Hospital, Capital Medical University, Beijing, China; ^2^ Department of Infectious Disease, Beijing Youan Hospital, Capital Medical University, Beijing, China; ^3^ Center for Clinical Epidemiology and Evidence-Based Medicine, Beijing Children’s Hospital, Capital Medical University, Beijing, China; ^4^ Beijing Institute of Hepatology, Beijing Youan Hospital, Capital Medical University, Beijing, China; ^5^ Department of Stomatology, Beijing Daxing District Hospital of Integrated Chinese and Western Medicine, Beijing, China; ^6^ Beijing Stomatological Hospital, Capital Medical University, Beijing, China

**Keywords:** saliva, virome, men who have sex with men, HIV, metagenomic analyses, metabolism

## Abstract

Salivary virome is important for oral ecosystem, but there are few reports on people living with HIV. We performed metagenomic sequencing to compare composition and functional genes of salivary virobiota between one HIV-negative and four HIV-positive groups in which participants were all men who have sex with men (MSM) with different immunosuppression statuses (five samples per group) to find the evidence that salivary virobiota plays a role in the pathogenesis of oral disease. Acute-stage subjects achieved a positive result of HIV RNA, but HIV antibody negative or indeterminate, whereas individuals with mild, moderate, and severe immunosuppression exhibited CD4^+^ T-lymphocyte counts of at least 500, 200–499, and less than 200 cells/μL or opportunistic infection, respectively. The results showed the composition of salivary virus genera in subjects with mild immunosuppression was the most similar to that in healthy people, followed by that in the acute stage; under severe immunosuppression, virus genera were suppressed and more similar to that under moderate immunosuppression. Furthermore, abnormally high abundance of *Lymphocryptovirus* was particularly obvious in MSM with HIV infection. Analysis of KEGG Pathway revealed that *Caulobacter* cell cycle, which affects cell duplication, became shorter in HIV-positive subjects. It is worth noting that in acute-stage participants, protein digestion and absorption related to the anti-HIV-1 activity of secretory leukocyte protease inhibitor was increased. Moreover, in the severely immunosuppressed subjects, glutathione metabolism, which is associated with the activation of lymphocytes, was enhanced. Nevertheless, the ecological dysbiosis in HIV-positive salivary virobiota possibly depended on the changes in blood viral load, and salivary dysfunction of MSM infected with HIV may be related to CD4 counts. Ribonucleoside diphosphate reductase subunit M1 in purine metabolism was negatively correlated, though weakly, to CD4 counts, which may be related to the promotion of HIV-1 DNA synthesis in peripheral blood lymphocytes. 7-Cyano-7-deazaguanine synthase in folate biosynthesis was weakly positively correlated with HIV viral load, suggesting that this compound was produced excessively to correct oral dysfunction for maintaining normal cell development. Despite the limited number of samples, the present study provided insight into the potential role of salivary virome in the oral function of HIV infected MSM.

## Introduction

Human oral cavity harbors thousands of microorganisms. Archaea, bacteria, eukaryota, and virus constitute an important ecological area of stable mutualism to guarantee oral health (1). Salivary viruses, which mostly comprised bacteriophages and herpesviruses, are regarded as reservoirs for pathogenic gene function; they are stable components of the oral ecosystem and play an essential role in maintaining the diversity of oral microbes (2, 3). However, unlike bacteria, the human virome components have not been fully identified, except for viruses that cause symptoms, such as human immunodeficiency virus (HIV) (4). Viruses have been ignored in the context of their effects on oral health, especially in people living with HIV (PLWH).

In previous studies, the diversity of salivary microorganisms (bacteria and fungi) in PLWH was investigated by16S rRNA and 18S rRNA analyses. There were significant changes in the alpha-diversity of oral bacteria in the saliva between HIV-infected and non-infected patients before receiving antiretroviral therapy (ART) (5–7). After ART, the overall difference in salivary bacteria between the HIV-positive and -negative individuals was small; changes in certain bacterial genera showed little effect on the diversity of the oral microbiome (8, 9), but there were significant differences in the oral fungal genera of HIV-positive individuals (10). With continuation of ART, the salivary bacterial community of HIV-positive individuals became more similar to that of HIV-negative individuals (11). Thus, research on the diversity of oral bacteria in PLWH has gradually matured.

Viral attack followed by replication affects the human immune system to varying degrees (12). Microbiome research on the local bacteria of patients with AIDS, as a chronic infectious disease, has gained attention; however, research on the viral components of the microbiome, termed virome, is lacking in comparison (13). In the past 10 years, only a few studies have focused on alterations in the enteric virome of HIV patients. A study using metagenomic analysis of the virome indicated pathogenic simian immunodeficiency virus (SIV) infection linked with expansion of the enteric virome (14). Further data suggested that low proportion of CD4^+^T lymphocytes was related to enteric adenovirus expansion (15). In SIV-infected gorillas, enteric virome potentially acts as a marker for lentiviral disease progression (16). Based on these findings in non-human primates, human virome may also show discernible differences after HIV infection (17). Therefore, virome changes in PLWH warrant further discussion.

Thus, in the present study, we performed metagenomic sequencing to compare the salivary virome composition of men who have sex with men (MSM) with HIV and the functional genes under different immunosuppressive statuses to find the evidence that salivary virobiota play a role in the pathogenesis of oral disease. Our findings on the relationship between HIV infection and virobiota in MSM infected with HIV will provide a new perspective for studies on oral microbiota in patients with immunosuppressive disorders.

## Methods

### Participants and Sample Collection

Study participants were all MSM from Beijing, China. The cross-sectional study was conducted under review and approval by the Institutional Review Board of Beijing Youan Hospital, Capital Medical University. All participants provided consent-written. The inclusion criteria included patients aged >18 years, not undergoing ART, untreated with immunomodulatory drugs, not taking antibiotics within the last 3 months, without history of any systemic disease, no oral problems (excluding non-cavitated caries, nonpurulent periodontal disease, and oral candidiasis), and having more than 20 teeth. The exclusion criteria included psychiatric condition, MSM partners, and inability to give informed consent. This trial protocol was registered at the Chinese Clinical Trial Registry (Identifier: ChiCTR2000030301).

Reference staging criteria of monitoring cases (18), 25 subjects screened strictly were evenly divided into four HIV-positive groups and one HIV-negative control group. The HIV-positive groups were classified according to disease stages as follows: stage 0, the early stage of HIV infection, i.e., a positive result of HIV RNA, but HIV antibody negative or indeterminate; stages 1, 2, and 3, CD4^+^ T-lymphocyte counts of at least 500, 200–499, and <200 cells/μL (opportunistic infection), respectively.

Participants were instructed not to eat, drink, or undergo oral hygiene procedures for at least 2 h prior to sample collection; from 10:00 to 13:00, the participants spit saliva (approximately 5 ml) into a 20 ml sterile tube, which was subsequently frozen at −80°C until DNA extraction (19).

### Metagenomic Sequencing and Annotation

Using the E.Z.N.A.^®^ Soil DNA Kit (Omega Bio-tek, Norcross, GA, U.S.), the total genomic DNA of salivary samples was extracted which was fragmented to an average size of about 400 bp by using Covaris M220 (Gene Company Limited, China) for paired-end library construction. Paired-end sequencing was performed by Majorbio Bio-Pharm Technology Co., Ltd. (Shanghai, China). Sequence data have been deposited in the NCBI (Accession Number: SRP327008). After removing adaptor sequences, trimming, and removing low-quality reads, the raw clean reads were mapped to the human hg38 reference genome using by BWA (http://bio-bwa.sourceforge.net, version 0.7.9a) (20) for identification and removal of the human host-originated reads. These high-quality reads were then assembled to contigs by using MEGAHIT (https://github.com/voutcn/megahit, version 1.1.2) (8). The open reading frames of contigs were identified by MetaGene (http://metagene.cb.k.u-tokyo.ac.jp/) (21), and a non-redundant gene catalog was constructed using CD-HIT (http://www.bioinformatics.org/cd-hit/, version 4.6.1) (22) to evaluate gene abundance in each sample with Reads Per Kilobase Million (RPKM) (23). According to Diamond (http://www.diamondsearch.org/index.php, version 0.8.35) (24), with e-value cutoff of 1e−5, representative sequences of non-redundant gene catalog and the KEGG were annotated.

### Statistical Analysis

SAS version 9.4 (SAS Institute Inc., Cary, NC, USA) was used for statistical analysis. Demographics were presented as medians and interquartile ranges, and comparisons between groups were performed by Kruskal–Wallis tests. Classification data were described in the form of numbers (percentages), and chi-square test was used for comparisons between groups. *P* value <0.05 was considered statistically significant.

LEfSe difference discriminant analysis was used to evaluate the differences in species, genes, and functions between the HIV-positive groups and the negative control group. Nonparametric factorial Kruskal–Wallis rank sum test was applied to detect significant differences in abundance. Wilcoxon rank sum test was conducted to test the consistency of the differences. Linear discriminant analysis (LDA) was performed to estimate the effect of the richness of species, genes, and functions on differences. Correlation heatmaps were generated by Spearman rank correlation analysis to visualize the relationships of viral KOs with CD4 counts and blood viral load (BVL). Significantly correlated KOs were annotated to metabolic pathways in the KEGG database: KOs ≥3 were compared between the HIV-positive groups by Wilcoxon rank sum test to identify differentially expressed enzymes.

## Results

### Participant Characteristics

We designed a cross-sectional study of meta-virome sequencing of the saliva of HIV-negative controls and untreated MSM with HIV infection. The demographic details are shown in **Table 1**. There was no significant difference in age, periodontal, or mucosal status between the five groups (*P >*0.05). Out of the five stage 3 patients enrolled, two had oral candidiasis. Moreover, there were significant differences (*P <*0.05) in CD4 count between four HIV-positive groups.

**Table 1:** Characteristics of participants in the untreated MSM with HIV infection and control groups.

Group	HIV_0	HIV_1	HIV_2	HIV_3	HIV_neg	Chi-	*P* value
	(n = 5)	(n = 5)	(n = 5)	(n = 5)	(n = 5)	square value	
Age, years	31	34	42	29	29	3.4527	0.4851
	(25.50, 43.00)	(24.50, 39.50)	(30.50, 46.00)	(22.50, 39.00)	(24.50, 39.00)		
Periodontal state, n (%)							
Healthy periodontal state	1 (20.00)	2 (40.00)	1 (20.00)	1 (20.00)	1 (20.00)	0.808	0.9374
Periodontitis	4 (80.00)	3 (60.00)	4 (80.00)	4 (80.00)	4 (80.00)		
Mucosal state, n (%)							
Healthy mucosal state	5 (100.00)	5 (100.00)	5 (100.00)	3 (70.00)	5 (100.00)	7.208	0.1253
Oral candidiasis	0 (0.00)	0 (0.00)	0 (0.00)	2 (30.00)	0 (0.00)		
CD4 count, cells/μL	354.33 (327.515, 470.52)	675.08 (604.52, 761.575)	243.00 (219.765, 374.725)	186.85 (63.44,234.04)	ND	15.7543	0.0013
BVL, log_10_ copies/ml	4.28	3.83	5.17	4.34	–	5.1792	0.1591
	(3.93, 4.94)	(3.47, 4.67)	(4.60, 5.465)	(3.965, 5.40)			

BVL, blood viral load; ND, Not detected; HIV_0, HIV-positive stage 0 group, HIV_1, HIV-positive stage 1 group, HIV_2, HIV-positive stage 2 group, HIV_3, HIV-positive stage 3 group, HIV_neg, HIV-negative control group.

Characteristics of participants are summarized using median with upper and lower quartiles or percentages and compared using Chi-square tests.

### Species Composition Characterization of the Saliva Virobiota

A total of 25 salivary samples were collected for virome sequencing using the Illumina NovaSeq platform, yielding 25,850,494 predicted gene sequences with a total length of 8,754,662,067 bp. After clustering, 100 non-redundant catalog genes were constructed, with an average length of 1,049.86 bp for each gene. The viral taxa from the saliva samples were annotated *via* comparison with the NR database as follows: 4 orders, 20 families, 79 genera, and 829 species.

**Figure 1:**
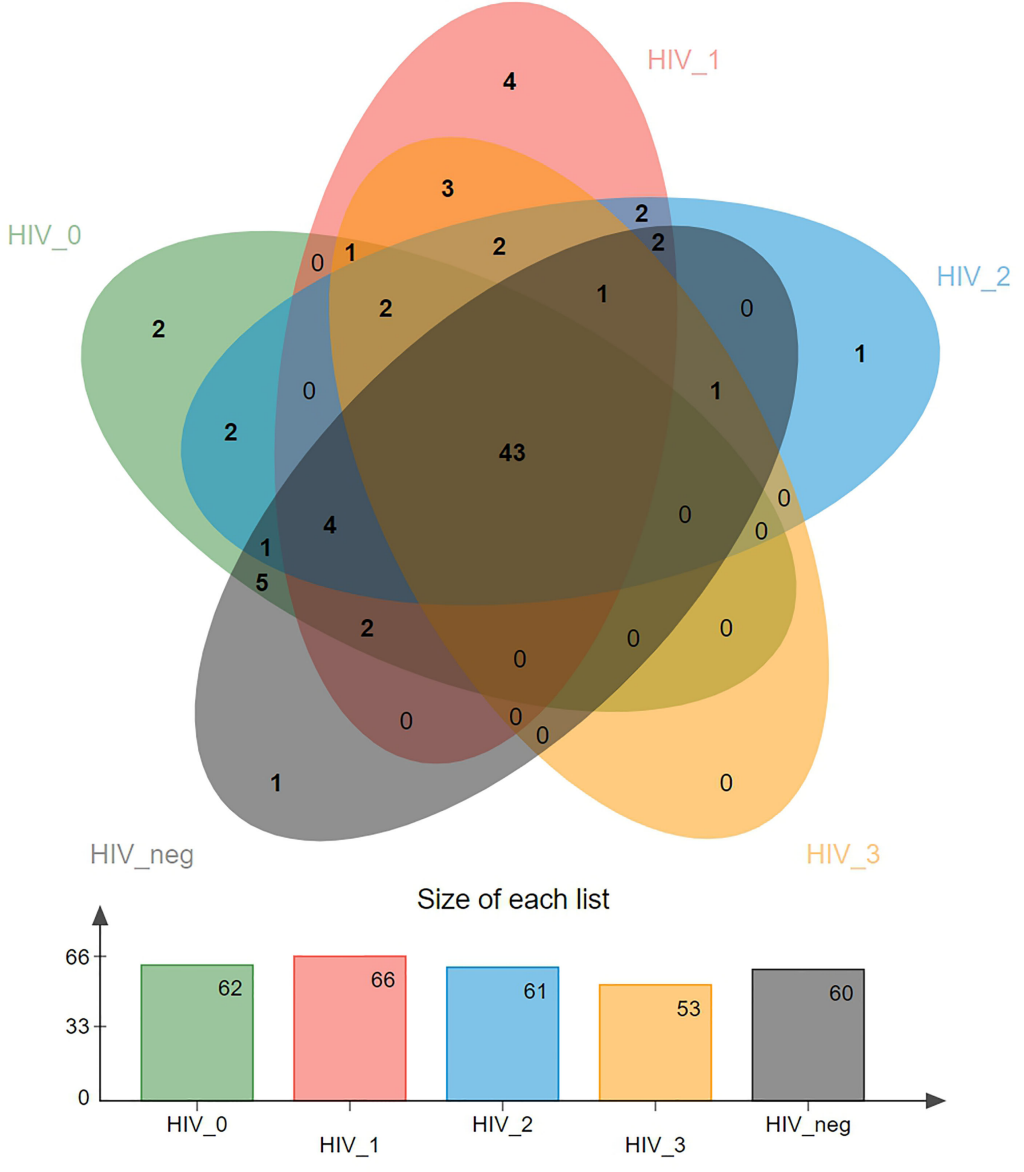
Venn diagram of the distribution of salivary viral genera in the five groups. Overlapping parts represent shared species, and non-overlapping parts represent unique species. The bar graph shows the total number of species in each group.

A Venn diagram showing differences in virome distribution between the five groups is presented in **Figure 1**. In total, there were 43 common genera in all groups, and each group had its own unique virus genera, except for the stage 3 group. There were two exclusive viruses in the stage 0 group, namely *Luz24likevirus* and *Felixounalikevirus*. *Epsilonretrovirus*, *Phifllikevirus*, *Spbetalikevirus*, and *Rhadinovirus* were only present in the stage 1 group. *Simplexvirus* and *Chilikevirus* were unique virus genera in the stage 2 and control groups, respectively.

**Figure 2:**
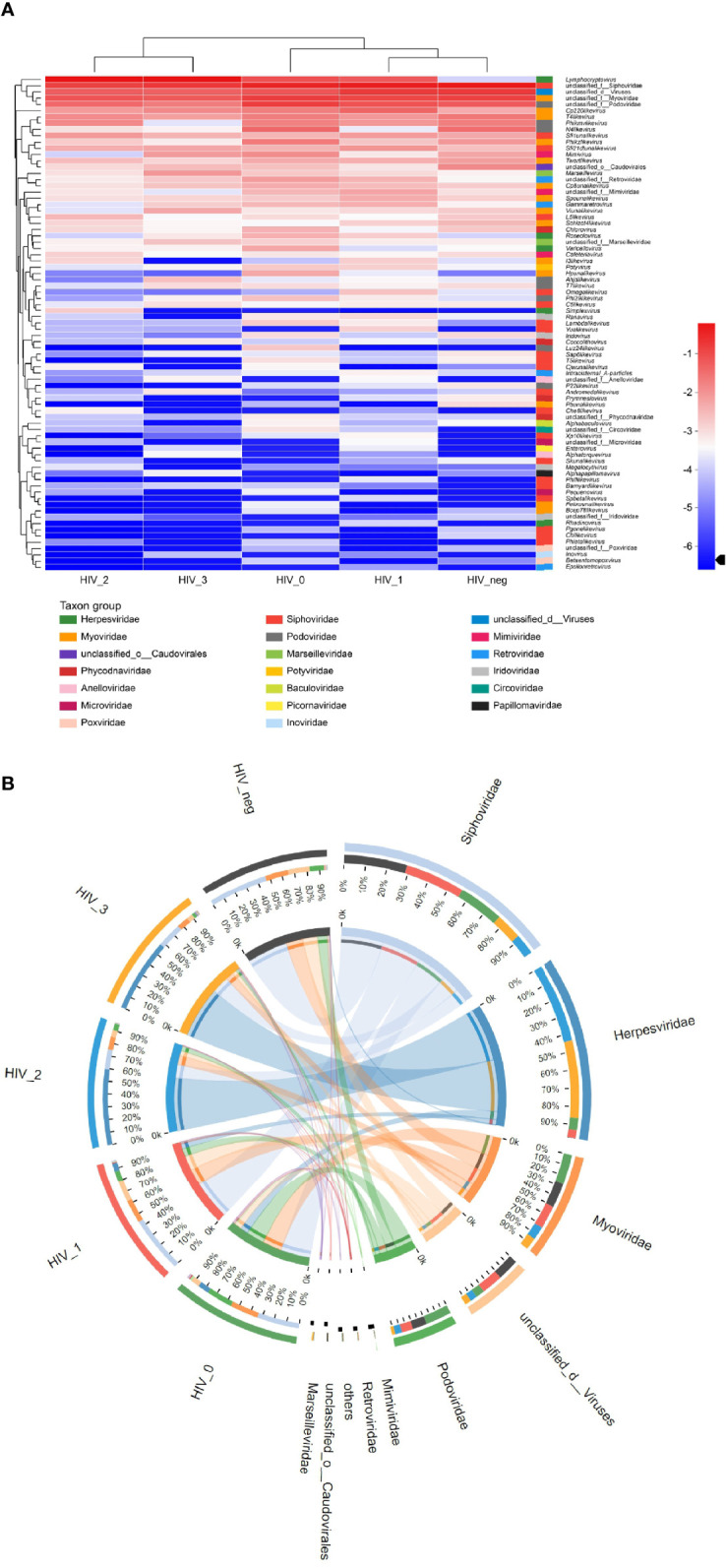
Community composition analysis diagram. **(A)** Heatmap visualizing the abundance of all viruses in saliva samples at the genus level. The left and upper sides are the species cluster tree and the sample cluster tree, respectively; the right and lower sides are different taxon groups of the family. Color intensity represents species abundance. **(B)** Circos diagram visualizing the abundance of viruses in saliva samples at the family level.

The composition of salivary virome in individuals of different disease stages was illustrated *via* heatmap (**Figure 2A**). We found that the five groups showed interesting clustering, in which the stage 1 group was more similar to the HIV-negative controls, whereas the stage 2 group was more similar to the stage 3 group. **Figure 2B** displays comprehensive comparison of virome composition between the five groups, the preponderance of virus families were Siphoviridae, Herpesviridae, Myoviridae, and Podoviridae in order. Most notably, *Lymphocryptovirus* of Herpesviridae was particularly rare in the HIV-negative group, but abundant in the HIV-positive groups.

### Differences in the Composition and Function of Saliva Virobiota

**Figure 3:**
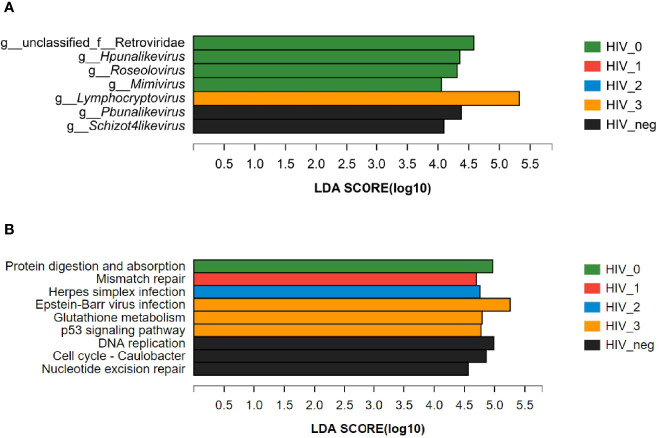
LEfSe bar charts of differences between the HIV-positive groups and HIV-negative controls. **(A)** Differences in salivary viral abundances; **(B)** Differences in the salivary viral functions at KEGG Pathway Level 3.

The LEfSe bar chart (**Figure 3A** **)** revealed the viruses with significant differences in abundance between the five groups, according to LDA. The unique *Lymphocryptovirus* (LDA value: 5.32441; *P* = 0.00859) was significantly enriched in the stage 3 group. Four viral taxa were more abundant in the stage 0 group, namely unclassified Retroviridae (LDA value: 4.58279; *P* = 0.01227), *Hpunalikevirus* (LDA value: 4.35265; *P* = 0.01666), *Roseolovirus* (LDA value: 4.31001; *P* = 0.01036), and *Mimivirus* (LDA value: 4.05485; *P* = 0.01391). The only viral genera that showed lower abundances in the HIV-positive groups were *Pbunalikevirus* (LDA value: 4.37982; *P* = 0.03503) and *Schizot4likevirus* (LDA value: 4.09366; *P* = 0.04403).

Using the KEGG PATHWAY database, the differences in the functions of salivary virome between the five groups were explored. LEfSe discriminant analysis of KEGG Pathway Level 3 (**Figure 3B**) revealed that the significantly enriched functions were protein digestion and absorption (LDA value: 4.97062; *P* = 0.02394) in the stage 0 group; mismatch repair (LDA value: 4.69967; *P* = 0.02326) in the stage 1 group; herpes simplex infection (LDA value: 4.7566; *P* = 0.00716) in the stage 2 group; Epstein–Barr virus infection (LDA value: 5.25784; *P* = 0.00683), glutathione metabolism (LDA value: 4.79322; *P* = 0.00791), and the p53 signaling pathway (LDA value: 4.77469; *P* = 0.01237) in the stage 3 group; DNA replication (LDA value: 4.98577; *P* = 0.00984), cell cycle—*Caulobacter* (LDA value: 4.86019; *P* = 0.00494), and nucleotide excision repair (LDA value: 4.56212; *P* = 0.01837) in the HIV-negative control group.

### Influence of CD4 Count and BVL on the Functions of Salivary Virus in MSM With HIV Infection

PERMANOVA was used to analyze the effect of different host factors on salivary virome. The results showed that CD4 count (R2: 0.10889; *P* = 0.034) was a significant factor affecting the functional variation at KEGG Pathway Level 3 of salivary virome in HIV-positive samples, apart from BVL (R2: 0.08186; *P* = 0.129). In addition, BVL (R2: 0.11225; *P* = 0.017) affected the changes in viral composition in all HIV-positive subjects.

**Figure 4:**
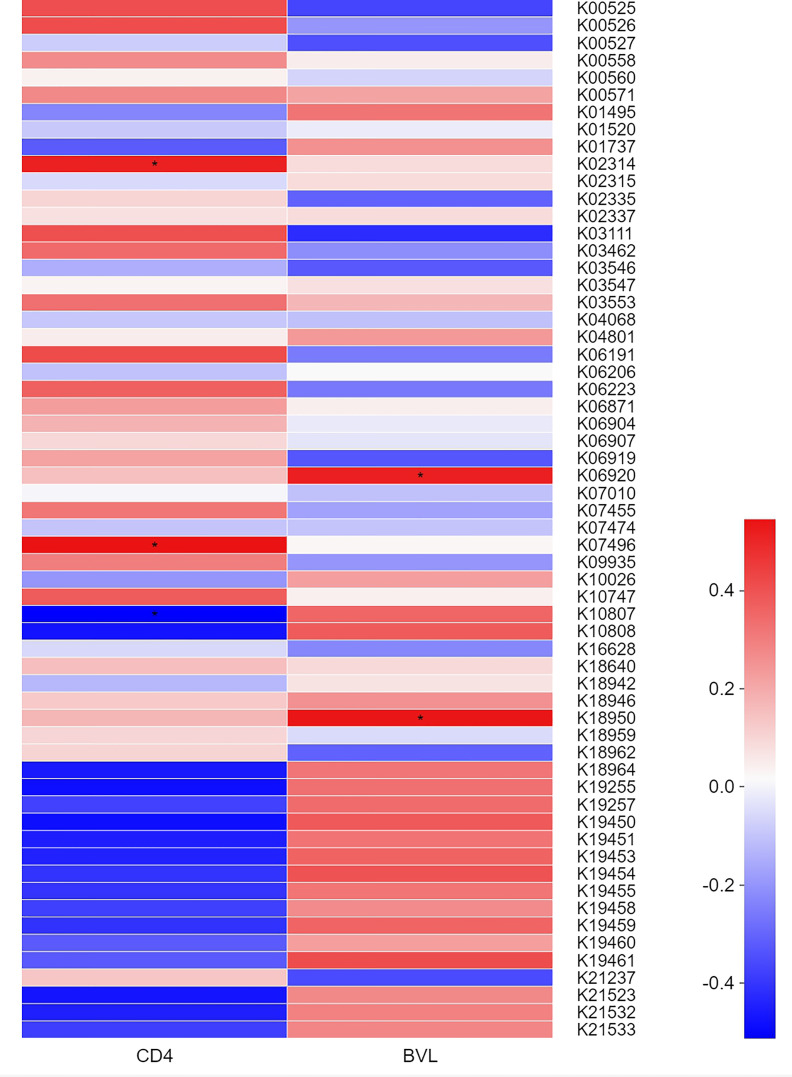
Correlation heatmap showing the relationships between the KOs of salivary virome of the top 60 abundance and BVL or CD4 counts in the four HIV-positive groups. Colors represent different r values (Spearman correlation coefficient): red and blue indicate positive and negative correlations, respectively. *0.01 <P ≤0.05.

Based on the KEGG Orthology (KO) classification system, heatmaps (**Figure 4**) were generated to identify KOs correlated with clinical indices. The top 60 most abundant KOs in samples from MSM participants were screened. In the four HIV-positive groups, K10807 was negatively but weakly correlated with CD4 count, whereas K02314 and K07496 showed the opposite trend. Moreover, K06920 and K18950 were significantly positively but weakly correlated with BVL. The detailed information of the functional genes was listed in **Table 2**.

**Table 2:** Significantly correlated KO genes and their functions in the four HIV-positive groups.

	KO	r value	*P* value	KO description	KEGG_Pathway	KEGG_Pathway description
CD4	K02314	0.51429	0.02035	Replicative DNA helicase [EC:3.6.4.12]	ko03030	DNA replication
					ko04112	Cell cycle—*Caulobacter*
	K07496	0.54532	0.01289	putative transposase	–	–
	K10807	−0.51246	0.02087	Ribonucleoside diphosphate reductase subunit M1 [EC:1.17.4.1]	ko00230	Purine metabolism
					ko00240	Pyrimidine metabolism
					ko00480	Glutathione metabolism
BVL	K06920	0.51502	0.02014	7-Cyano-7-deazaguanine synthase [EC:6.3.4.20]	ko00790	Folate biosynthesis
	K18950	0.53870	0.01426	Ribonuclease H [EC:3.1.26.4]	–	–

KO, KEGG Orthology; EC, Enzyme naming and numbering of Enzyme Commission of IUB; ko, pathway of KOs.

**Figure 5:**
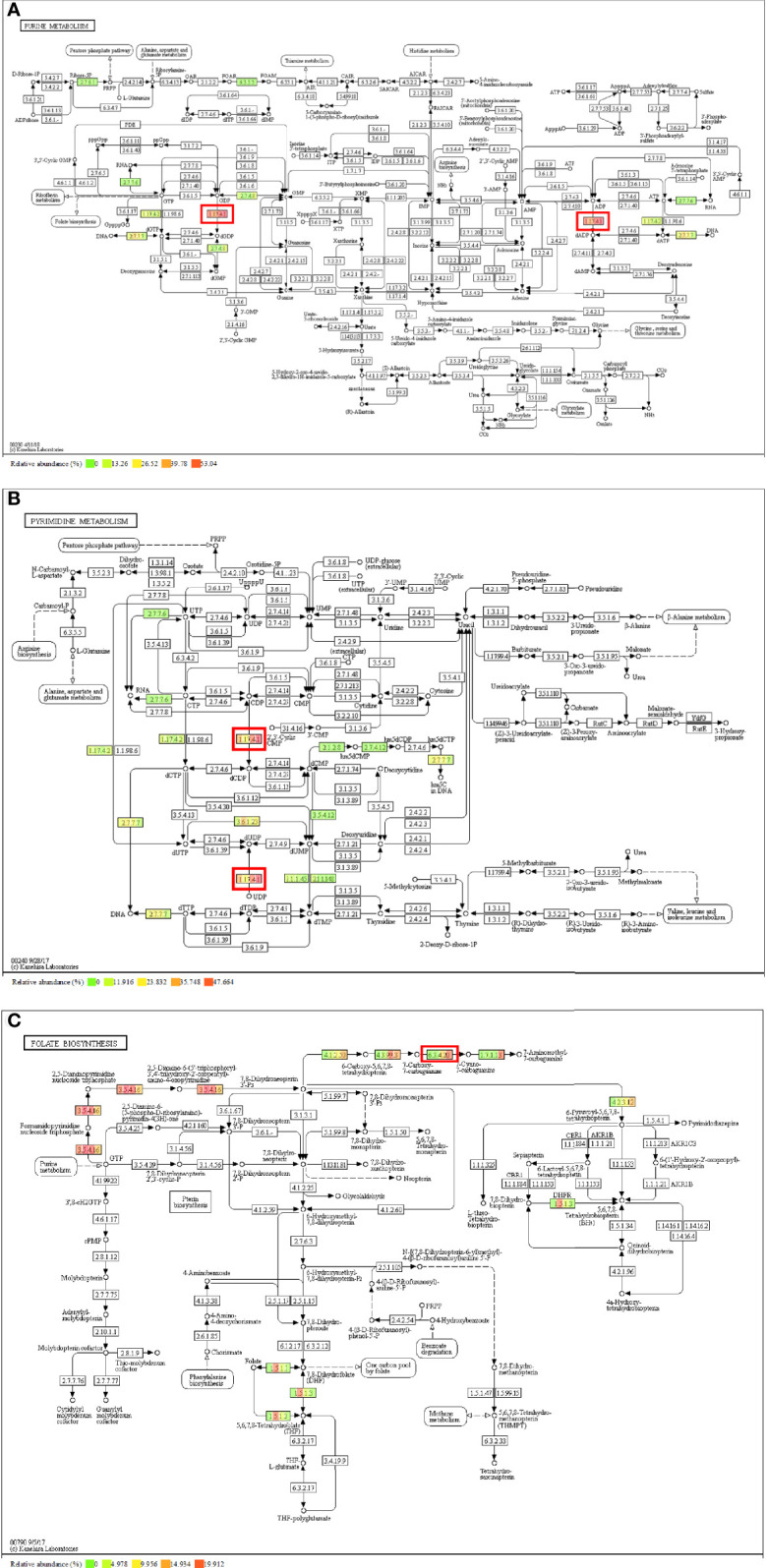
Metabolism pathway and annotated enzyme of the correlated KO genes. **(A)** Purine metabolism and [EC:1.17.4.1]; **(B)** Pyrimidine metabolism and [EC:1.17.4.1]; **(C)** Folate biosynthesis and [EC:6.3.4.20].

We explored the differences between the four HIV-positive groups for KOs ≥3 metabolic pathways-purine metabolism, pyrimidine metabolism, and folate biosynthesis—where the related KOs were located, and observed the corresponding enzymes that regulate the metabolic functions in different pathways. Annotated ribonucleoside diphosphate reductase subunit M1 (RRM1) [EC:1.17.4.1] were shown abundance variations between four HIV-positive groups and the roles which participated in the conversion of GDP to dGDP and ADP to dADP in purine metabolism (**Figure 5A**) and the conversion of CDP to dCDP and UDP to dUDP in pyrimidine metabolism (**Figure 5B**). However, that enzyme was affected by CD4 counts, performing a negative correlation. Likewise, in folate biosynthesis (**Figure 5C**), the annotated 7-cyano-7-deazaguanine synthase [EC:6.3.4.20] played a vital role in the conversion of 7-carboxy-7-carbaguanine to 7-cyano-7-carbaguanine, and it was positively affected by BVL.

## Discussion

Here, we conducted a cross-sectional study comparing the salivary virobiota of untreated HIV-infected patients according to the disease stages. Although this is a new perspective, it was conducted to further the study of the changes in salivary bacterial community with the disease progression of HIV infection (7). With metagenomic analysis, which offers a high power of decision making (25), differences in the composition and function of salivary virobiota between the five groups were revealed, indicating that they may be closely related to the oral mucosal lesions of MSM with HIV infection.

The current results also established that MSM infected with HIV exhibited similar virobiota distribution to the HIV-negative population, with 43 viruses shared between the five groups and only few unique viruses in each group. We initially thought that there would be many unique viruses in the virome of AIDS patients, but there were none. Instead, patients with mild immunosuppression stage exhibited the highest variety of genera, indicating that salivary virus diversity at the genus level may also partially decrease under severe immunosuppression, but increase under mild immunosuppression. Previous studies have determined Siphoviridae, Myoviridae, and Podoviridae as the most abundant virus families in saliva of HIV-negative individuals (26, 27), which was consistent with our findings of salivary virome composition in the HIV-negative group. In addition, we found a significant increase in the abundance of *Lymphocryptovirus* of Herpesviridae in saliva samples from HIV-positive participants, especially in the stage 3 group. Interestingly, oral transmission has been confirmed as a common route of *Lymphocryptovirus* infection (28). Although there has been no clinical evidence that *Lymphocryptovirus* can induce oral or maxillofacial tumors in PLWH, experiment in rhesus models of simian immunodeficiency virus (SHIV) have shown that *Lymphocryptovirus* promotes oncogenesis and induces the development of malignant lymphoma in the immunodeficient hosts (29–31), verifying the high abundance of *Lymphocryptovirus* in the 3 stage group in the present study. Therefore, the abnormally high abundance of *Lymphocryptovirus* in the HIV-positive groups is worthy of attention, warranting an in-depth investigation.

Sample cluster tree analysis revealed that the virobiota of the stage 1 group was the most similar to that of the controls, followed by that of the stage 0 group; in contrast, the virobiota of the stage 2 group was more similar to that of the stage 3 group. We speculate that the variation in saliva virome was caused by the following process: (1) when HIV invades the host body, virome dysbiosis is initiated because of acute infection; (2) following the production of HIV antibodies, HIV and the host undergo symbiosis, and virome composition in saliva tends to be normal; (3) once CD4^+^T lymphocytes are significantly depleted (<500 cells/μL) to the moderate or severe immunosuppressive state, the virome structure will be markedly different from that in the healthy state.

LEfSe discriminant analysis identified the unique genera and gene functions in the different stages of HIV disease progression. We observed interesting changes in salivary viral taxa between HIV-positive and -negative participants. Individuals in the acute stage exhibited the most abnormal abundance of viral taxa, but had the least functional alterations. It is possible that the high proportion of Retroviridae promoted protein digestion and absorption, suggesting that HIV infection enhanced the synthesis and release of proteases by salivary virome. Previous studies (32–34) have confirmed that saliva contains secretory leukocyte protease inhibitor (SLPI), which can inhibit HIV-1 activity, especially in the early infection stage, that is, the body produces protease as a protective mechanism against the virus to maintain oral functions. In the asymptomatic stage, instead of abnormally increasing in abundance, the structure of salivary virobiota tends to stabilize; nevertheless, mismatch repair (MMR) and herpes simplex infection begin to develop as immunity declines. MMR enrichment under mild immunosuppression indicates the compensatory initiation of body-related functions, and the lack of MMR proteins may stimulate tumor mutation in HIV-infected persons (35). In the moderate immunosuppressive state, the oral virome function is unbalanced again as herpes simplex infection progresses in the oral keratinized tissue, hard palate, or gums, resulting in vesicles that recurrently rupture to form painful ulcers (36). However, Epstein–Barr virus infection may enhance glutathione (GHS) metabolism and the p53 signaling pathway, indicating that imbalance of the virome function in the severe immunosuppressive state is aggravated. This is highly detrimental because (1) AIDS patients are at a high risk of oral viral infection, and saliva is the main route of EBV transmission, which can cause disruption in periodontal infections, oral hairy leukoplakia, and various related lymphoid and epithelial malignancies (37–39); (2) The deficiency of GHS, which affects lymphocyte activation, may provoke oxidative stress or impair the antioxidant system, thus contributing to clinical manifestations of HIV (40, 41). Hence, we presume that the excessive GHS metabolism of salivary virome might represent GHS exhaustion; (3) As a pro-apoptotic transcription factor, p53 can further aggravate the neuronal damage induced by HIV (42, 43) and participate in HIV-induced cell cycle arrest and apoptosis (44). A previous study (45) in an *in vitro* model of HIV latency revealed that inhibition of the p53 signaling pathway might increase the proportion of CD4^+^T cells, in line with our current findings. In general, in the salivary virobiota of MSM with HIV infection, *Caulobacter* cell cycle becomes shorter, and DNA replication and nucleotide excision repair become weaker, disrupting the duplication of cells (46) and repair of damaged cells (47).

The ecological dysbiosis detected in the saliva virobiota of MSM subjects was most probably related to the BVL variation, whereas the saliva dysfunction of MSM with HIV infection might be due to changes in the CD4 count. KO analysis results revealed significantly enriched viral genes in HIV infected MSM: ribonucleoside diphosphate reductase subunit, which is a key enzyme in DNA synthesis and plays an important role in nucleic acid metabolism; RRM1, which is involved in the synthesis of dCDP and dUDP in the purine metabolism of salivary viruses, and tends to gradually aggravate as the immune system is depleted. This may be attributed to the high expression of deoxyribonucleotides catalyzed by the ribonucleoside diphosphate reductase subunit in cells, which can promote HIV-1 DNA synthesis in peripheral blood lymphocytes (48). As we know, HIV invades host cells through the high-affinity binding of gp120 and the CD4 receptor, which results in the destruction of the host immune system. However, the potent replicative ability of HIV affects cell cycle arrest and cell apoptosis by regulating changes in protein expression (49), which is similar to our research findings. We also observed that in folate biosynthesis, which is involved in cell growth and reproduction, 7-cyano-7-deazaguanine synthase level increased as HIV replication increased, thus promoting the catalysis of 7-carboxy-7-carbaguanine into 7-cyano-7-carbaguanine. Furthermore, it is possible that salivary viruses excessively replicate to maintain the normal development of cells as compensation for the imbalance of the host oral function. Therefore, we propose that the activity of HIV affects protease activity in saliva to certain degrees. However, owing to the lack of studies, metabolic pathways in the saliva of MSM infected with HIV, such as purine and pyrimidine metabolism pathways, are not fully understood. Nevertheless, it is certain that the oral metabolites of MSM with HIV infection are different from those of healthy people, and that changes in amino acid levels may be closely related to opportunistic infections (50).

Our study has a few limitations. First, since the small sample size, the true of salivary virome in MSM infected with HIV may be substantially different. Second, the cross-sectional study is based on the viewpoint of rapid ART after HIV infection (51–54) and ethical considerations. It is impossible to establish cohort of patients who did not receive ART at different stages of infection. Finally, there are many factors affecting oral microbiological research, such as smoking, periodontal disease, and dental caries. Non-strict controls of the oral environment of MSM participants could lead to biased results.

## Conclusion

In conclusion, metagenomic analysis revealed that the composition and function of salivary virobiota in MSM infected with HIV differed according to the disease stages. The virobiota may play a certain role in regulating metabolic balance and host immunity, suggesting a close relation between variations in salivary virobiota characteristics and the BVL of HIV, whereas functional imbalance of salivary virome was related to AIDS disease progression. Although this study had a limited number of samples, we showed the potential effect of salivary virome on oral health in MSM with HIV infection. These findings are preliminary results, and we will increase the sample size for verification in further studies and explore more potential associated factors, such as ART, gender.

## Data Availability Statement

The datasets presented in this study can be found in online repositories. The names of the repository and accession number can be found in the article.

## Ethics Statement

The studies involving human participants were reviewed and approved by the Institutional Review Board of Beijing Youan Hospital, Capital Medical University. The patients/participants provided their written informed consent to participate in this study.

## Author Contributions

YG, XH, and ZS led the analysis and writing of the manuscript. YG, XH, FZ, DZ, and ZS contributed to the final version. XS, YY,YW, BZ, JC, SW, HD, YL, XW, WX, FW, JD, and SG were involved in managing the data collection. SC was responsible for data analysis. All authors contributed to the article and approved the submitted version.

## Funding

This work was supported by the National Science and Technology Major Project of China during the 13th Five-year Plan Period (grant no. 2017ZX10201101), the Beijing Excellent Talent Plan (grant no. 2018000021223ZK04), the Beijing Talent Project in the New Millennium (grant no. 2020A35), and the National Natural Science Foundation of China (grant no. 81701984).

## Conflict of Interest

The authors declare that the research was conducted in the absence of any commercial or financial relationships that could be construed as a potential conflict of interest.

## Publisher’s Note

All claims expressed in this article are solely those of the authors and do not necessarily represent those of their affiliated organizations, or those of the publisher, the editors and the reviewers. Any product that may be evaluated in this article, or claim that may be made by its manufacturer, is not guaranteed or endorsed by the publisher.
